# Immune Alterations in a Patient With Hyperornithinemia-Hyperammonemia-Homocitrullinuria Syndrome: A Case Report

**DOI:** 10.3389/fimmu.2022.861516

**Published:** 2022-05-27

**Authors:** Silene M. Silvera-Ruiz, Corinne Gemperle, Natalia Peano, Valentina Olivero, Adriana Becerra, Johannes Häberle, Adriana Gruppi, Laura E. Larovere, Ruben D. Motrich

**Affiliations:** ^1^Centro de Investigaciones en Bioquímica Clínica e Inmunología (CIBICI-CONICET), Facultad de Ciencias Químicas, Universidad Nacional de Córdoba, Córdoba, Argentina; ^2^Centro de Estudio de las Metabolopatías Congénitas (CEMECO), Hospital de Niños de la Santísima Trinidad, Facultad de Ciencias Médicas, Universidad Nacional de Córdoba, Córdoba, Argentina; ^3^Division of Metabolism and Children’s Research Center, University Children’s Hospital Zurich, Zurich, Switzerland; ^4^Fundación para el Progreso de la Medicina, Córdoba, Argentina; ^5^División de Enfermedades Metabólicas, Hospital de Niños de la Santísima Trinidad, Córdoba, Argentina; ^6^Cátedra de Clínica Pediátrica, Facultad de Ciencias Médicas, Universidad Nacional de Córdoba, Córdoba, Argentina

**Keywords:** urea cycle defects, HHH syndrome, infection, hyperammonemia, immunodeficiency, T cells, B cells, case report

## Abstract

The hyperornithinemia-hyperammonemia-homocitrullinuria (HHH) syndrome is a rare autosomal recessive inborn error of the urea cycle caused by mutations in the *SLC25A15* gene. Besides the well-known metabolic complications, patients often present intercurrent infections associated with acute hyperammonemia and metabolic decompensation. However, it is currently unknown whether intercurrent infections are associated with immunological alterations besides the known metabolic imbalances. Herein, we describe the case of a 3-years-old girl affected by the HHH syndrome caused by two novel *SLC25A15* gene mutations associated with immune phenotypic and functional alterations. She was admitted to the hospital with an episode of recurrent otitis, somnolence, confusion, and lethargy. Laboratory tests revealed severe hyperammonemia, elevated serum levels of liver transaminases, hemostasis alterations, hyperglutaminemia and strikingly increased orotic aciduria. Noteworthy, serum protein electrophoresis showed a reduction in the gamma globulin fraction. Direct sequencing of the *SLC25A15* gene revealed two heterozygous non-conservative substitutions in the exon 5: c.649G>A (p.Gly217Arg) and c.706A>G (p.Arg236Gly). *In silico* analysis indicated that both mutations significantly impair protein structure and function and are consistent with the patient clinical status confirming the diagnosis of HHH syndrome. In addition, the immune analysis revealed reduced levels of serum IgG and striking phenotypic and functional alterations in the T and B cell immune compartments. Our study has identified two non-previously described mutations in the *SLC25A15* gene underlying the HHH syndrome. Moreover, we are reporting for the first time functional and phenotypic immunologic alterations in this rare inborn error of metabolism that would render the patient immunocompromised and might be related to the high frequency of intercurrent infections observed in patients bearing urea cycle disorders. Our results point out the importance of a comprehensive analysis to gain further insights into the underlying pathophysiology of the disease that would allow better patient care and quality of life.

## Introduction

The mammalian urea cycle provides a pathway for the synthesis of the non-essential amino acid arginine and serves to detoxify ammonia, keeping plasma ammonium concentrations within a narrow range despite ten-fold variations in dietary nitrogen intake (1, 2). The mitochondrial transporter ornithine carrier 1 (ORC1) plays a crucial role in the cycle by transferring cytosolic ornithine into the mitochondrial matrix in exchange for citrulline (3, 4). Mutations in the solute carrier family 25 member 15 (*SLC25A15*) gene, which encodes for ORC1, are causative of the rare autosomal recessive urea cycle disorder (UCD) called hyperornithinemia–hyperammonemia–homocitrullinuria (HHH) syndrome (OMIM 238970) (1, 5). In affected patients, ORC1 deficiency reduces the rate of the urea cycle leading to hyperammonemia, a typical feature of most UCD (1, 6, 7). A founder effect was reported in the French-Canadian population (1) and then 50 affecting function variants have been already identified in 122 patients worldwide pointing out the diversity of mutations and pan-ethnic distribution (8–10). The disease usually manifests in early infancy or childhood; however, cases of adult onset have also been reported (6, 11, 12). Typical clinical features include lethargy, episodic confusion or coma due to postprandial hyperammonemia, hepatitis-like vomiting, spastic paraplegia, cerebellar ataxia, seizures, failure to thrive, coagulation factor defects, and liver failure (1, 7, 12). Different precipitants such as dietary carelessness, enhanced protein catabolism consequent to dietary over-restriction or infections may trigger an acute deterioration of the metabolic status, which is characterized by potentially life-threatening episodes of hyperammonemia (13), which may lead to cerebral edema, lethargy, anorexia, vomiting, hyperventilation (or hypoventilation), hypothermia, neurologic posturing, coma and death. Interestingly, intercurrent infections are often observed in UCD patients and have been reported as the most frequent precipitant of acute hyperammonemia episodes and metabolic decompensation (13, 14). Furthermore, they are the most dangerous precipitant since the induced inflammation is a catabolic stressor that usually aggravates the patient clinical status significantly increasing morbidity and mortality risks (13, 15–17). Although a considerable advance in the knowledge about the pathophysiology of UCD and their neurologic and metabolic consequences has been achieved during recent years, very scarce data about their putative consequences on the immune system have been reported up to date (12, 17–19). Certainly, it is currently unknown whether the frequently observed intercurrent infections are associated with putative immune alterations, which could render patients susceptible to infections further increasing the severity of the hyperammonemia episodes and thus resulting in higher hospitalization rates and longer hospital stays (13, 17).

Herein, we report the case of a patient with HHH syndrome caused by two novel mutations in the *SLC25A15* gene associated with recurrent otitis episodes that precipitated hyperammonemia crises. These non-previously reported mutations consisted of two missense mutations in exon 5 of the *SLC25A15* gene, namely c.649G>A (p.Gly217Arg) and c.706A>G (p.Arg236Gly). Interestingly, the patient showed functional and phenotypic immune alterations that could render her immunocompromised and thus explaining her history of recurrent otitis.

## Case Description

**Figure 1:**
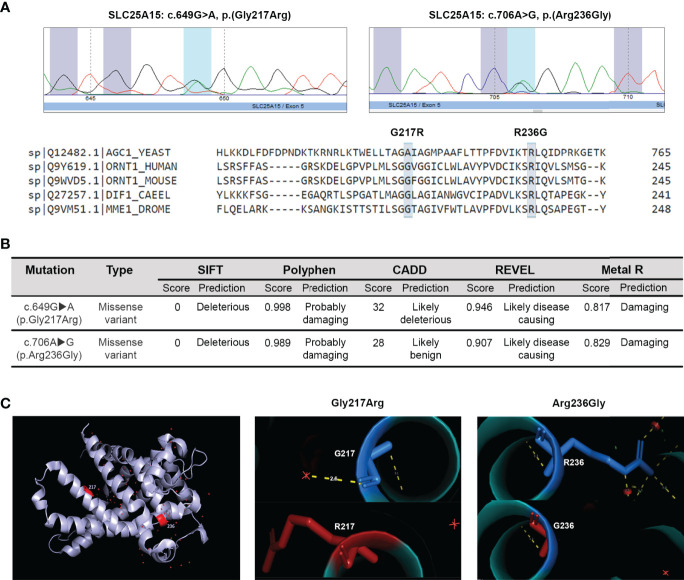
*In silico analysis of c.649G>A (p.Gly217Arg) and c.706A>G (p.Arg236Gly) mutations on the ORC1 protein structure and function*. **(A)** Partial electropherograms showing the detected mutations. Multiple alignment of the modified ORC1 aminoacid residues with the protein sequence from different species using the Clustal W2 software. **(B)** *In silico* validation of the two *SLC25A15* gene mutations identified. **(C)** Modeling of the tridimensional protein structural changes introduced by the two identified mutations using the human ORC1 homology model. Normal residues are marked in blue (top) and the changes introduced by mutations in red (bottom).

We present the case of a girl aged 3.4 years, who was admitted to the emergency room on August 5, 2019, for recurrent acute otitis with vomiting, intermittent sensory disturbance, and irritability. She is the second child of healthy non-consanguineous Caucasian parents. She was born at term by spontaneous delivery with a weight of 3500 g and no history of perinatal complications or developmental delay. Interestingly, her parents report that 11 months earlier (September 2018) the child was admitted into another hospital because of a similar episode of acute infectious otitis with somnolence, confusion, and lethargy. When the patient was examined in our hospital, the neurological assessment found her sleepy but reactive to stimuli, with no neurologic deterioration (Glasgow coma score of 15/15), and normal brain computed tomography scan. As shown in **Table 1**, the patient presented with severe hyperammonemia, increased serum levels of aspartate aminotransferase (AST) and alanine aminotransferase (ALT), metabolic alkalosis, and markedly reduced prothrombin activity and prolonged aPTT. Analysis of amino acids in serum revealed increased levels of glutamine. Furthermore, urine analysis revealed strikingly increased orotic acid excretion. Noteworthy, blood cytology showed increased proportions and counts of monocytes and atypical lymphocytes (20) (**Table 1**), probably a consequence of the ongoing otitis episode. Besides, and remarkably, serum protein electrophoresis revealed low levels of total proteins mainly due to decreased levels of the gamma globulins fraction indicating a possible underproduction of antibodies. Interestingly, reduced levels of IgG and slightly increased levels of IgM were detected (**Table 1**). Owing to the severe hyperammonemia and increased orotic aciduria, the patient was suspected to suffer from a UCD, more specifically a case of ornithine transcarbamylase deficiency (OTCD) or HHH syndrome. An OTCD was excluded by genetic analysis, which revealed no mutations in the *OTC* gene. However, direct sequencing of the *SLC25A15* gene identified two novel heterozygous mutations in exon 5 of the *SLC25A15* gene (ENST00000338625.9): c.649G>A (p.Gly217Arg) and c.706A>G (p.Arg236Gly) (**Figure 1A**), different from the 50 already reported *SLC25A15* gene mutations (5, 8–10), strongly suggesting a case of HHH syndrome.

**Table 1:** Laboratory findings in a patient with HHH syndrome.

		At admission	At relapse (18 months later)	Reference values^*^
Plasma ammonia (μmol/L)		**360**	**230**	≤ 40
Plasma aminoacids	Citrulline	33	–	0-50
(μmol/L)	Ornithine	113	**436**	0-250
	Ornithine/citrulline ratio	3.5	–	1.5-20.0
	Arginine	31	–	0-100
	Glutamine	**1346**	**1154**	333-809
	Glutamic acid	238	142	0-600
Metabolite urinary excretion	Orotic acid (μmol/mmol creatinine)	**684.5**	–	< 10.0
Liver enzymes	aspartate aminotransferase (AST)	**70**	**36**	5-25
(U/L)	alanine aminotransferase (ALT)	**160**	**40**	3-25
	alkaline phosphatase (ALP)	303	**696**	70-448
	gamma glutamyl transferase (GGT)	30	14	5-39
Hemostasis	Prothrombin activity (%)	**42**	–	80-100
	aPTT (seg)	**94**	–	28-46
Blood cytology	Red blood cell count (x 10^6^/μL)	4.51	4.85	4.00-5.20
	Hematocrit (%)	38.5	38.5	33.0-42.5
	Hemoglobin (g/dL)	12.1	12.3	11.0-14.2
	Leukocyte cell count (x 10^3^/μL)	9.54	11.87	5.50-15.50
	Neutrophils [/μL, (%)]	3530 (37)	2968 (**25**)	1500-7300 (27-50)
	Eosinophils [/μL, (%)]	191 (2)	237 (2)	0-500 (0-3)
	Basophils [/μL, (%)]	0 (0)	0 (0)	0-100 (0-2)
	Lymphocytes [/μL, (%)]	4293 (**45**)	7834 (**66**)	2300-8000 (50-56)
	Monocytes [/μL, (%)]	**1145** (**12**)	356 (3)	0-900 (0-5)
	Atypical lymphocytes [/μL, (%)]	**286** (**3**)	**475** (**4**)	0-100 (0-1)
Lymphocyte subsets	NK cells [/μL, (%)]	–	260 (**3.3**)	246-461 (6.0-14.0)
(flow cytometry)	B cells [/μL, (%)]	–	671 (**8.6**)	411-685 (11.0-18.0)
	T cells [/μL, (%)]	–	**5664** (72)	2054-3169 (67.0-75.0)
	CD4+ T cells [/μL, (%)]	–	**4452** (**78.6**)	1129-1581 (33.0-43.5)
	CD8+ T cells [/μL, (%)]	–	**1212** (**21.4**)	711-1121 (22.5-29.5)
	CD4:CD8	–	**3.67**	1.12-1.93
Serum protein	Total proteins (g/dL)	**6.3**	6.62	6.4-8.3
electrophoresis	Albumin [g/dL, (%)]	3.89 (61.8)	4.03 (60.9)	3.85-4.83 (55.0-69.0)
	Alpha-1 globulin [g/dL, (%)]	0.27 (4.3)	0.17 (2.5)	0.07-0.42 (1.0-6.0)
	Alpha-2 globulin [g/dL, (%)]	0.74 (11.8)	0.77 (11.7)	0.42-0.84 (6.0-12.0)
	Beta-1 globulin [g/dL, (%)]	0.44 (7.0)	0.71 (10.7)	0.42-0.84 (6.0-12.0)
	Beta-2 globulin [g/dL, (%)]	0.33 (5.3)	0.19 (2.8)	0.07-0.21 (1.0-3.0)
	Gamma globulin [g/dL, (%)]	**0.62** (**9.8**)	**0.75** (11.4)	0.77-1.26 (11.0-18.00)
	Albumin/Globulin	1.62	1.56	1.00-2.00
Serum	IgM (mg/dL)	**109**	**126**	38-90
immunoglobulins	IgG (mg/dL)	**608**	**690**	701-1157
	IgE (UI/mL)	32	17	< 90
	IgA (mg/dL)	112	80	66-120

^*^Reference normal values according to patient age and sex currently established and used in the local setting (Hospital de Pediatría Dr. Juan P. Garrahan, Buenos Aires, Argentina). Bold values mean abnormal or out of the reference range values.

The putative structural and functional consequences of the newly identified *SLC25A15* gene missense mutations were assessed *in silico*. The mitochondrial ORC1 transporter is composed of 301 amino acids with six transmembrane segments. Arginine 217 resides in the fifth transmembrane domain, and Glycine 236 is located in an alpha helix along the substrate translocation pore. As shown in **Figure 1A**, multiple sequence alignment analysis revealed that G217R and R236G are both non-conservative substitutions in amino acid residues evolutionarily conserved among species, which suggests they are crucial for protein structure and function. Moreover, different bioinformatics prediction tools such as Clustal W (21), SIFT (22), Polyphen (23), CADD (24), REVEL (25) and Metal R (26) indicated that both mutations are highly detrimental for the secondary structure and function of the protein since both substituted amino acids differ in polarity, charge, size, and other biochemical properties (**Figure 1B**). The majority of the single-residue mutations reported causing the HHH syndrome are non-conservative substitutions that introduce changes in the charge and size of the amino acid side chain (5, 27). Moreover, computer modelling and mapping of both missense mutations using the 3D structural homology model of ORC1 (28) indicated that residue Arg236 is located in the matrix gate area and plays a crucial role in conformational changes which allow substrate translocation (29). Conversely, the residue Gly217 belongs to the Pro-Gly level 1 immediately above the substrate binding region (30), playing the crucial hinge/kink function, which allows conformational changes and the access of the substrate to the ORC1 binding site (**Figure 1C**). The above reported observations indicate that both mutations have a high likelihood to impair ornithine translocation, coherently with the patient clinical phenotype supporting/confirming a diagnosis of HHH syndrome.

After the initial diagnostic work-up, the patient was treated with intravenous sodium benzoate, sodium phenylacetate, and L-arginine that rapidly normalized plasma ammonia levels in less than 24 hours. Immediately after, the patient was put on a protein restricted diet (1.3 g/kg/day) supplemented with L-carnitine (100 mg/kg/day), L-arginine (100 mg/kg/day), and sodium benzoate (230 mg/kg/day) that resulted in metabolic correction, remarkable clinical and biochemical improvement, without any complications or sequelae. In addition, the otitis episode resolved after amoxicillin treatment (80 mg/kg/day) for 7 days. However, 18 months later (February 2021) the patient relapsed and was re-admitted to the hospital with a similar clinical picture as at first admission: an episode of recurrent otitis associated to irritability, intermittent sensory disturbance, and confusion. Once again, severe hyperammonemia accompanied with increased serum levels of ornithine, glutamine, AST, ALT and also alkaline phosphatase (ALP) were detected. Interestingly, blood cytology revealed increased proportions of lymphocytes and elevated counts of atypical lymphocytes once more (**Table 1**). Moreover, serum protein electrophoresis consistently revealed reduced levels of the gamma globulins fraction of serum proteins. In addition, reduced levels of IgG and increased levels of IgM were detected again (**Table 1**). Taking into consideration these data and the patient history, we then performed a comprehensive phenotypic and functional analysis of cellular and humoral adaptive immune compartments once the patient was metabolically compensated and released from the hospital.

**Figure 2:**
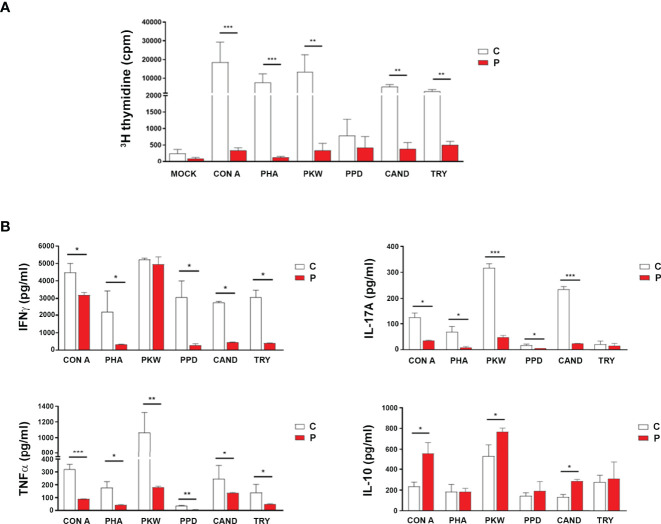
*Functional analysis of cellular adaptive immune compartment*. **(A)** Lymphoproliferative responses of peripheral blood mononuclear cells (PBMC) from the patient (P) and control (C) individuals to different mitogenic stimuli [concanavalin A (Con A), phytohemagglutinin (PHA), Pokeweed (PKW), candidin, and antigens of *Mycobacterium tuberculosis* or *Trychophyton* spp.] or medium alone (mock). Cells were cultured (3.0x10^5^/well) in quadruplicate for each condition and incubated for 96 h at 37°C/5% CO_2_ as previously described (33). During the last 18 h of culture, wells were pulsed with 1 µCi of [methyl-3H] thymidine in fresh medium. Cells were harvested onto glass fiber filters and labeled material was counted in a β scintillation counter. Results were expressed as counts per minute (cpm). **(B)** IFNγ, IL-17A, TNF-α and IL-10 concentrations in PBMCs culture supernatants determined by sandwich ELISA and results expressed as pg/ml. Experiments were performed at least in quadruplicate and repeated twice with similar results. Data are shown as mean ± SEM, the patient and sex and age-matched control individuals (n=6 healthy females, aged 3-7 years old). Red bars correspond to the average values of the patient and white bars correspond to the average values of the controls analyzed. Mann-Whitney test; *p< 0.05, **p< 0.01 and ***p< 0.005.

**Figure 3:**
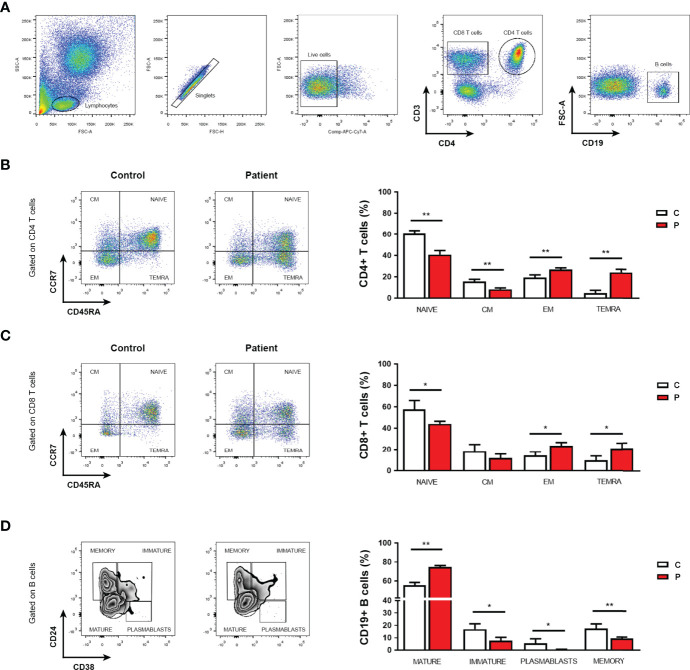
*Phenotypic analysis of T and B cells*. **(A)** Flow cytometry gating strategy for the assessment of phenotypic cell markers in live CD4 and CD8 T cells, and B cells from PBMC after red blood cell depletion by lysing buffer treatment. **(B, C)** Frequencies of naïve T cells, central memory (CM), effector memory (EM), and terminally differentiated effector memory cells (TEMRA) within the CD4+ **(B)** or CD8+ **(C)** T cell populations from the patient (P) under study and controls (C). **(D)** Frequencies of mature, immature and memory B cells, and plasmablasts in peripheral blood from the patient (P) under study and controls (C). Representative flow cytometry dot plots. Experiments were performed in triplicates. Data are shown as mean ± SEM, the patient and sex and age-matched control individuals (n=6 healthy females, aged 3-7 years old). Red bars correspond to the average values of the patient and white bars correspond to the average values of the controls analyzed. Mann-Whitney test; *p< 0.05 and **p< 0.01. The following fluorescent-labeled anti-human antibodies (BioLegend) were used: CD3 (PerCP), CD4 (APC), CD19 (APC), CD45RA (PE-Cy7), CCR7 (PE), CD24 (FITC) and CD38 (PE). Dead cells were excluded using LIVE-DEAD™ fixable (Invitrogen). Data were collected on FACS-CANTO II flow cytometer (BD Biosciences) and analyzed using FlowJo software (version 7.6.2). Proper compensation using Fluorescence Minus One (FMO) controls were used.

Immunocompromised patients usually present a profound immune dysregulation associated with increased morbidity and mortality, which has been characterized by reduced *in vitro* lymphocyte proliferative capacity in response to mitogens and alterations in the production of pro-inflammatory cytokines (31, 32). Therefore, proliferative responses from peripheral blood mononuclear cell (PBMC) from the patient and sex and age-matched healthy control individuals to well-defined mitogens including concanavalin A (Con A), phytohemagglutinin (PHA), Pokeweed (PKW), candidin, and antigens of *Mycobacterium tuberculosis* or *Trychophyton* spp. were assessed. As shown in **Figure 2A**, significantly reduced proliferation rates in response to most of the mitogens assayed were observed in the patient with respect to controls. When assaying the production of inflammatory cytokines, significantly reduced levels of IFNγ, IL-17A and TNFα secretion were detected in culture supernatants of PBMC from the patient compared with controls (**Figure 2B**). Conversely, significantly higher levels of the IL-10 were detected in culture supernatants from the patient PBMC stimulated with Con A, PKW, and candidin with respect to controls (**Figure 2B**). In parallel, we quantified the levels of CD4+ and CD8+ T cells, B cells and NK cells in the patient peripheral blood by flow cytometry. Whereas normal counts of NK and B cells were observed, significantly higher counts of T cells were detected, mainly due to an increase in the CD4+ T cell subset (**Table 1**). These findings indicated an expansion of that particular T cell population and were in agreement with the increased levels of atypical lymphocytes revealed by blood cytology (**Table 1**). Considering that, we then evaluated the distribution of CD4+ and CD8+ T cell subsets in the patient under study and sex and age-matched healthy control individuals. CD45RA and CCR7 cell surface markers were used to identify four phenotypically and functionally distinct subsets of CD4+ and CD8+ T-cells: naïve (Naïve: CD45RA+ CCR7+), central memory (CM: CD45RA- CCR7+), effector memory (EM: CD45RA- CCR7-) and terminally differentiated effector memory (TEMRA: CD45RA+ CCR7-) (**Figures 3A–C**) as previously reported (34–36). **Figure 3B** shows that, within the CD4+ T-cell compartment, the frequency of naïve and CM T cells were significantly lower in the patient than in controls (p<0.01). In contrast, the patient revealed significantly higher proportions of EM and TEMRA CD4+ T cells than control individuals (p<0.01, **Figure 3B**). When analyzing the CD8+ T-cell compartment, significantly reduced naïve and increased EM and TEMRA CD8+ T cells were respectively observed in the patient with respect to controls (**Figure 3C**). These results indicated alterations in the T cell compartment from the patient under study.

As serum protein electrophoresis analyses revealed low levels of gamma globulins, which was further confirmed by the detection of low levels of serum IgG, we then analyzed B cell subsets in peripheral blood by flow cytometry. Although total B cell counts were within reference values, significantly reduced frequencies of plasmablasts, immature and memory B cells were found in the patient with respect to sex and age-matched healthy controls (**Figure 3D**). Conversely, the patient revealed significantly higher frequencies of mature B cells than control individuals (**Figure 3D**). These data revealed that the patient had reduced counts of circulating plasmablasts and memory B cells that could be related to the detected hypogammaglobulinemia and low levels of serum IgG but increased IgM. Altogether, the results show alterations in the adaptive T and B cell immune compartments that would render the HHH patient immunocompromised and might explain her history of recurrent otitis episodes.

## Discussion

Several inborn errors of metabolism affecting effectors of the urea cycle or UCD have already been described in humans, including the HHH syndrome (12, 18). Although hyperammonemia and related complications are the main and best described consequences of the HHH syndrome and other UCD, little is known about other putative consequences on different tissues and cells such as those from the immune system.

Herein, we are reporting a case of a girl that was admitted to the hospital with an episode of recurrent otitis together with signs and symptoms compatible with a UCD. Due to the clinical presentation, laboratory findings, and local prevalence (37), an OTCD was initially suspected. However, sequencing of the *OTC* gene revealed no alterations excluding OTCD as the underlying pathology. Based on its prevalence, we then suspected a case of HHH syndrome even though the metabolic triad that usually defines the HHH syndrome, hyperammonemia, hyperornithinemia, and urinary excretion of homocitrulline was not observed in the patient at admission. Nevertheless, genetic analysis revealed two novel heterozygous mutations in *trans* of the *SLC25A15* gene that *in silico* analysis further indicated to likely impair the functionality of the transporter protein thus confirming a diagnosis of HHH syndrome. These results support previously reported data by Mahmoud et al., 2019, who predicted several possible HHH syndrome causative mutations (38). Interestingly, the R236G mutation is listed in the Single Nucleotide Polymorphism Database (dbSNP; rs142236568) with an allele frequency of 0.02% but it has never been reported as causative of the HHH syndrome or any other disease.

The patient presented hyperammonemic crises after recurrent infectious otitis episodes, which was not surprising since intercurrent infections are commonly observed in UCD patients (13, 15, 16). As it has been shown that the inability to ingest food and medications did not seem to be associated with infection, and cannot readily explain the observed increasing morbidity in UCD patients (13), the pathophysiology of intercurrent infections could be due in part to immune alterations. The assessment of the T cell compartment revealed increased counts of T cells, mainly due to an increase in the CD4+ T helper cell subset. Interestingly, higher levels of effector memory cells and low levels of naïve cells were found in both CD4+ and CD8+ T cell populations. Strikingly, the patient showed significantly increased levels of terminally differentiated effector memory (TEMRA) CD4+ and CD8+ T cells. These results mirrored the dynamics of T cell changes associated with T cell dysfunction typically observed in the elderly and cytomegalovirus infected patients (39–42). Indeed, TEMRA is a hallmark of cellular senescence, including reduced proliferation and defective mitochondrial function (43, 44). In agreement, the patients showed strikingly reduced PBMC proliferative responses to polyclonal stimuli or memory recall antigens associated with reduced secretion of IFNγ, IL-17A, and TNFα, and increased secretion of IL-10. These results indicated an altered lymphocyte proliferation state with reduced ability to produce pro-inflammatory cytokines and enhanced production of immunoregulatory cytokines, a phenotype compatible with immunosuppression (31, 32, 45–47). On the other hand, the patient showed hypogammaglobulinemia and reduced serum IgG but increased IgM levels both at first admission and after relapsing 18 months later. Noteworthy, when assessing B cell subpopulations in peripheral blood, the patient showed strikingly reduced levels of circulating plasmablasts and memory cells but increased frequencies of mature cells suggesting a possible impairment in the activation and differentiation of B cells, and/or an inability to sustain the survival of the former B cell subpopulations. Altogether, these results show alterations in the adaptive T and B cell immune compartments that would render the HHH patient immunocompromised thus explaining recurrent infections.

Although no immune alterations have been reported in HHH syndrome or other UCDs so far, our results suggest they might be present in patients bearing these rare inborn errors of metabolism, which is a still overlooked area in medical research and clinical practice. In fact, our results could be related to the well-known high frequency of intercurrent infections typically observed in these patients. Interestingly, Monaco *et al.* revealed that the *SLC25A15* gene is expressed by most leukocyte subpopulations with plasmablasts and CD4+ T cells showing the highest levels (48, 49). Although the specific implications of defective *SLC25A15* gene expression in these cells still need to be unveiled, our results suggest that a defective expression of this gene might alter B and T cell function paving the way for future research in the area. In that regard, argininosuccinate synthetase 1 (ASS1), another component of the urea cycle that when defective causes citrullinemia type I (OMIM# 215700), is also expressed in several organs and cells of the immune system. Interestingly, experimental models have shown that the deficiency of ASS1 leads to abnormal T cell differentiation and function despite normal hepatic expression (50). Moreover, it has been shown that some patients bearing lysinuric protein intolerance, another rare hereditary disorder that secondarily affects the urea cycle and manifests with hyperammonemia, showed decreased levels of serum IgG sub-classes and suboptimal vaccine responses pointing to a B-cell dysfunction (51).

In agreement with our study, these reported data further suggest that in addition to metabolic disturbances, UCD patients may have impaired immune responses. Besides the putative consequences of the deficiency of components of the urea cycle in immune cells, it has been shown that hyperammonemia *per se* also affects immune cell function. The *in vitro* exposure to high levels of ammonia impairs neutrophil and dendritic cell phagocytic function and reduces the ability of the latter to induce lymphocyte activation and proliferation (52, 53). Moreover, cirrhotic patients, who are usually hyperammonemic and metabolically decompensated although to a lesser degree than UCD patients, are susceptible to opportunistic infections. Noteworthy, impaired phagocytic neutrophil function, aberrant immunoglobulin glycosylation, and increased exhaust CD8+ T cells have been found in cirrhotic patients indicating a state of immunocompromise associated with hyperammonemia (54–57). A limitation of this study is that the levels of different leukocyte subsets and T and B cell subpopulations from the patient at admission and at different times during her evolution are unknown. In that regard, it would be interesting to assess if correction of hyperammonemia correlates with improvements in T and B cell number and function. In that regard, we believe that the identified immune alterations herein may also be occurring in other metabolic diseases where ammonia is a common factor. Future studies may help confirm the hypothesis that stricter ammonia control is required in these patients, probably much lower than current recommendations (<80 μmol/L) (12), which could improve the overall health status of patients beyond just avoiding neurotoxicity. In addition, although the *in silico* analyses indicated that both mutations detected would very likely affect the activity of the ORC1 transporter, being compatible with the patient clinical phenotype, the definitive pathogenicity of both mutations should be demonstrated by experimental models *in vitro* and/or *in vivo* designed *ad hoc*.

In conclusion, our study identified two non-previously described mutations in the *SLC25A15* gene underlying HHH syndrome. Moreover, we are reporting for the first time functional and phenotypic immunologic alterations in this rare inborn error of metabolism that would render the patient immunocompromised and might be related to the high frequency of intercurrent infections typically observed in UCD patients. Our results highlight the need of a more comprehensive evaluation of patients and warrant further investigation of the putative implications of the HHH syndrome and other UCD on the immune system, which will allow a better understanding of these rare diseases and thus a better patient care and quality of life.

## Patient Perspective

The patient has remained stable and with no further complications after adhering to the treatment proposed. Family was satisfied with the improvement in the clinical condition, has been committed to maintain medical care at home and regularly attend to periodic controls.

## Data Availability Statement

The original contributions presented in the study are included in the article/supplementary material, further inquiries can be directed to the corresponding author.

## Ethics Statement

The studies involving human participants were reviewed and approved by Institutional Review Board of the Hospital de Niños de la Santísima Trinidad, Facultad de Ciencias Médicas, Universidad Nacional de Córdoba. Written informed consent to participate in this study was provided by the participants’ legal guardian/next of kin. Written informed consent was obtained from the minor(s)’ legal guardian/next of kin for the publication of any potentially identifiable images or data included in this article.

## Author Contributions

RM and LL: conceptualization, funding acquisition, resources, supervision, data analysis and curation, writing and editing of the manuscript. SR-R: data acquisition, formal analysis, *in silico* analysis, validation, writing or the manuscript. CG, NP, VO and JH: methodology, data acquisition. AB: patient care. AG: resources, intellectual content, revision of the manuscript. All authors reviewed the manuscript. All authors significantly contributed to the article and approved the submitted version.

## Funding

This work was supported by the Agencia Nacional de Promoción Científica y Tecnológica (ANPCyT-FONCyT, grant PICT 2014-2195 and 2019-2451), CONICET (grant PIP0100652) and Secretaría de Ciencia y Tecnología de la Universidad Nacional de Córdoba (Secyt-UNC).

## Conflict of Interest

The authors declare that the research was conducted in the absence of any commercial or financial relationships that could be construed as a potential conflict of interest.

## Publisher’s Note

All claims expressed in this article are solely those of the authors and do not necessarily represent those of their affiliated organizations, or those of the publisher, the editors and the reviewers. Any product that may be evaluated in this article, or claim that may be made by its manufacturer, is not guaranteed or endorsed by the publisher.

## Abbreviations

HHH, hyperornithinemia–hyperammonemia–homocitrullinuria; ORC1, ornithine carrier 1; UCD, urea cycle disorder/s; OTCD, ornithine transcarbamylase deficiency; CM, central memory; EM, effector memory; TEMRA, terminally differentiated T effector memory cells that re-express the naïve T cell-associated marker CD45RA.
